# A novel detection method for wheat aging based on the delayed luminescence

**DOI:** 10.1038/s41598-024-51563-0

**Published:** 2024-01-11

**Authors:** Gong Yue-hong, Liu Yu-kun, Gong Zhi-le, Zhong Xiao-yan, Zhao Wei-ting, Li Bing, Ge Hong-yi, Lyu Qiong-shuai

**Affiliations:** 1 School of Software, Pingdingshan University, Pingdingshan, 467000 China; 2 Henan International Joint Laboratory for Multidimensional Topology and Carcinogenic Characteristics Analysis of Atmospheric Particulate Matter PM2.5, Pingdingshan, 467000 China; 3 School of Computer and Software, Pingdingshan Polytechnic College, Pingdingshan, 467000 China; 4 School of Information Science and Engineering, Henan University of Technology, Zhengzhou, 450001 China

**Keywords:** Biological techniques, Computational biology and bioinformatics

## Abstract

Wheat aging plays an important role in assessing storage wheat quality and its subsequent processing purposes. The conventional detection methods for wheat aging are mainly involved in chemical techniques, which are time-consuming as well as waste part of wheat samples for each detection. Although some physical detection methods have obtained gratifying results, it is extremely hard to expand their application fields but to stay in the theory stage. For this reason, a novel nondestructive detection model for wheat aging based on the delayed luminescence (DL) has been proposed in this paper. Specifically, after collecting enough sample data, we first took advantage of certain hyperbolic function to fit DL signal, and then used four parameters of the hyperbolic function to feature the decay trend of the DL signal. Secondly, in order to better feature the DL signal, we extracted other six features together with above four features to form the input feature vector. Finally, as the bidirectional long short-term memory (Bi-LSTM) network lacked error-correcting performance, the Bi-LSTM network based on Walsh coding (Walsh-Bi-LSTM) mechanism was proposed to establish the detection model, which made the detection model have the error-correcting performance by reasonably splitting the multi-classification target task. Shown by experimental results, the newly proposed wheat aging detection model is able to achieve 94.00% accuracy in the testing dataset, which can be used as a green and nondestructive method to timely reflect wheat aging states.

## Introduction

Wheat plays an important role in strategic grain reserve in the world by virtue of its excellent storage characteristics. Wheat kernels, as a type of organism, will still carry on respiration function to maintain their lives after harvest^1^. With time elapsing, wheat kernels continue consuming their own energy and then cause wheat aging, which not only deteriorates the quality, but also wastes the quantity of storage wheat^2^.

The main physiological changes of wheat aging were characterized by losing various enzymatic activities and destroying membrane integrality, impairing RNA and protein synthesis, and so forth^3^. Besides the physiological changes mentioned above, it was illustrated that the germination rate of wheat kernels drastically decreased, while the fatty acid value and malonaldehyde content obviously increased after artificial aging^4^. It was shown that the viscosity value and the falling value of wheat kernels would be increased with prolongation of the storage time, which were two disadvantage factors for wheat storage^5,6^. Therefore, the study on wheat aging degree detection is of great importance for ensuring the quality and the quantity of storage wheat.

Generally, conventional detecting techniques for wheat aging mainly rely on chemical methods, such as guaiacol method, triphenyl tetrazolium chloride method, and acidity indicator method. Specifically, guaiacol method is an indirect detection technique to reflect the wheat aging degree via measuring the characteristics of peroxidase which widely exists in animals, plants, and microbes. Wang et al.^7^ realized the paddy freshness degree detection using guaiacol method. The principle of triphenyl tetrazolium chloride method is that the embryo colour of vigorous wheat kernels can be dyed to red after certain reactive process provided that dehydrogenase of inner embryo cell absorbs the water thoroughly. Yang et al.^8^ successfully applied triphenyl tetrazolium chloride method into detecting wheat aging degree. Acidity indicator method refers to detecting the wheat aging degree based on the regularity of fatty acid value of wheat kernels. Based on the acidity indicator method, Zhao et al.^9^ used Methyl Red and Bromothymol Blue as an acid indicator to show the relationship between paddy aging degree and its storage period. To these conventional chemical methods, however, they are always involved in long pretreatment processes to wheat samples and causing certain quantity waste of wheat samples due to their destructive detection ways. Furthermore, traditional chemical methods may pollute the environment caused by the chemical reagents used in the whole detection process. Meanwhile, these chemical methods mainly rely on one index to detect the wheat aging degree, whose common bottleneck lies in lacking a way to comprehensively reflect the real life state of wheat kernels.

In order to better solve the inherent defects of chemical methods, a multitude of physical methods have been proposed for wheat aging detection, such as electronic nose technique^10^, near infrared spectrum technique^11^, terahertz technique^12^ and so on. However, to electronic nose technique, the main problem is that the mechanism of electronic nose is complicated and the baselines of sensors are easy to drift. Therefore, sensitivity and stability of the sensors in electronic nose system need to be improved. Although the near infrared spectrum technique and terahertz technique have achieved accurate detection degree to wheat aging, both of them are involved in using large equipment, and can only carry on detection in the laboratory rather than on site.

With the development of electrooptical technology and the emergence of photomultiplier, an ocean of experiments have validated biophoton emission is a common phenomenon to all the organisms in nature. In fact, biophotons derive from non-local coherent electromagnetic fields within living matter, which can provide a comprehensive indicator to reflect the basic properties of biological system as well as a novel method to detect wheat aging degree. Generally, biophoton emission, also named as ultra weak luminescence (UWL), consists of spontaneous luminescence (SL) and DL. The SL of organism is a kind of “ullage” and carries information of living system during this process. The DL of organism is that the tested sample will show a certain relaxation phenomenon after being induced by the white or other monochromatic illuminants. Compared with SL signals, DL signals are prone to have a higher Signal to Noise Ratio (SNR) and be collected in a more efficient way. Meanwhile, a striking progress in DL research domain was achieved.

Early in 1923, Russian biologists Gurwitsch^13^ used biological detectors to test the roots of onion and found a special phenomenon that onion cells produced weak light which stimulated other cells to accelerate division while performing cell division. The scientist named Colli took advantage of photomultiplier to detect the photon radiation intensity of wheat, bean and maize distributing from 250 counts/(s cm^2^) to 700 counts/(s cm^2^), and the range of their spectrum is among 400–700 nm^14^. The initial DL phenomenon was observed by Strehler and Arnold^15^, when they detected algae samples for ATP formation by the induced luminescence. Subsequently, the DL phenomena were discovered in other biological samples, such as green plant^16^, luminous bacteria research^17^, living organisms^18^, and so on. Up to now, DL detection technique has been widely applied in food quality evaluation^19^, milk samples analysis^20^, Chinese herbal materials research^21^, and so on. To wheat samples, since the SNR of DL signals are much higher than that of SL signals, detecting wheat aging degree based on the DL signals is more convenient and feasible.

## Materials and methods

### Sample preparation and instrument parameter configuration

Above all, all the following experiments on wheat samples were carried out according to the institutional guidelines and legislation. Wheat samples in five different years from 2015 to 2019 (we successively use SW2015–SW2019 to label them in the following chapters) were offered by Suiping Grain Bureau, Zhumadian City, Henan province, China. Firstly, we picked out cracking kernels and other foreign materials, and then washed them three times by distilled water. Secondly, we took advantage of the electric blast drying oven to dry the samples till the moisture was (12.5 ± 0.2)%. Thirdly, each type of wheat was prepared about three kilograms and put these five types wheat into five valve bags marked by corresponding year for the subsequent measurements.

**Figure 1:**
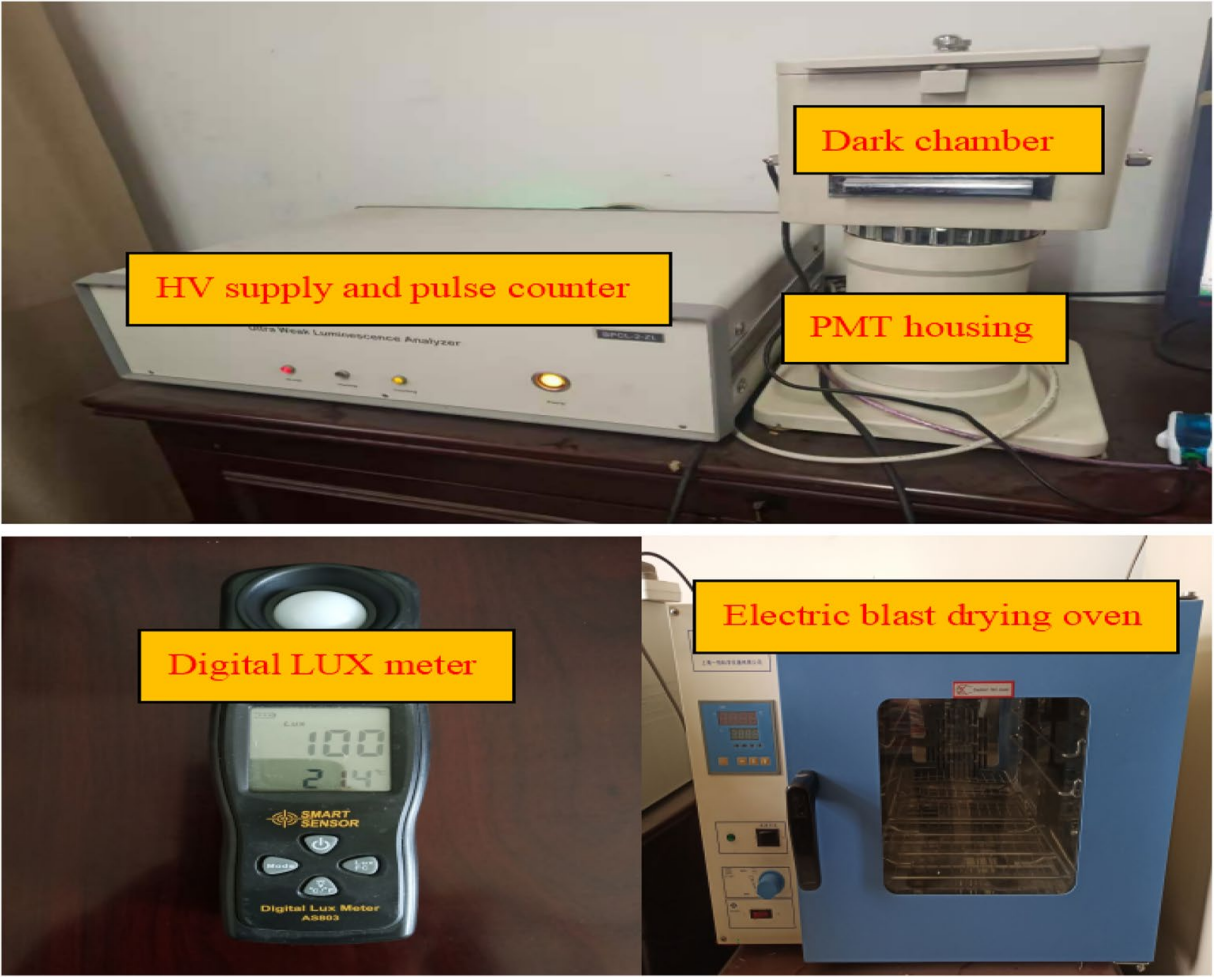
The main instruments used in this work.

The type of UWL analyzer is BPCL-2-ZL, manufactured by Beijing Jianxin Lituo Technology CO., LTD, which can be seen in Fig. 1. The UWL analyzer consists of three parts: ① dark chamber, where we put the tested wheat samples and fix a cold white Light-Emitting Diode (LED) as the induced illuminant; ② photomultiplier (PMT) housing, which is used for changing photon signals into electrical signals; ③ high voltage (HV) supply and pulse counter, taking advantage of the pulse amplitude screening technology and the digital counting function to collect the photon signals.

The parameters of UWL analyzer are:

- testing spectral range: 300–650 nm
- fastest sampling rate: 0.1 ms
- peak voltage output: 1500 V
- operating conditions: voltage (220 V, 50 Hz); temperature (5–40 °C); relative humidity (20–80%)

In the following tests, measurement temperature and HV of the UWL analyzer is set as (20.0 ± 0.5) °C and 1030 V respectively.

The type of Electric Blast Drying Oven is DHG-9030A, manufactured by Shanghai Yiheng Instruments CO., LTD. Working conditions: voltage (220 V/50 Hz); temperature (10–250 °C).

The type of Digital Lux Meter is AS803, manufactured by Hong Kong Smart Instrument Group CO., LTD. Testing range: 1 LUX ~ 200,000 LUX; operating temperature: − 10–60 °C; measurement error: ± 2%; resolution: 1 LUX. LUX stands for the unite of intensity of illumination. Figure 1 shows the main instruments used in this work.

Compared with other induced illuminants, LED is finally chosen in this work because of the following advantages:

- LED belongs to a type of all-solid, anti-seismic, long service life, small single power cold illuminant, and can still work even under low voltage conditions.
- LED shows better monochromatic and steady optical properties, and the light intensity is not easily affected by voltage fluctuations.
- LED has fast response characteristics, and can timely achieve continuous work states without preheating.
- The volume of LED is small, which is easy for further design and installing in a small place like the dark chamber.

The type of LED is YLF5WOS1-3097, offered by Dongguan Yingyue Electronic Technology Co., Ltd. Operation parameters: colour temperate (2900–3100 K), K is the unit of Kelvin; voltage (3.0 V); power (0.06 W); luminous intensity (1650–2640 mcd), mcd is the abbreviation of Micro Candela.

### DL signal acquisition

First of all, before carrying out the experiment, keeping the same ambient conditions, such as, indoor temperature at (20 ± 1) °C, relative humidity at (21 ± 6)%, testing time (8:00 a.m.–6:30 p.m.) and so on, is of necessity for the sake of minimizing the influences caused by environmental factors. In addition, we randomly select certain wheat samples to test the analyzer so as to choose the optimum working parameters, and setting the following parameters after many tests: measuring temperature is 20 °C; high voltage is 1030 V; sampling interval is 0.1 s; and the total measuring time for each wheat sample is 100 s. Finally, the empty chamber has been measured several times according to the above-mention parameters in order to obtain the average background noise of the analyzer.

**Figure 2:**
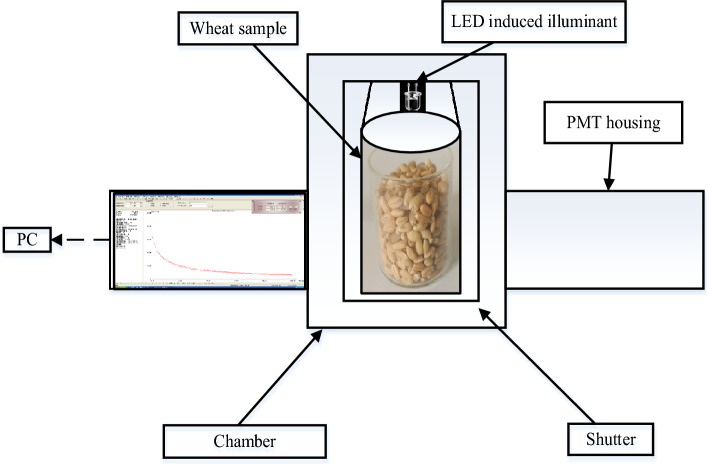
The schematic diagram of measuring DL signal of wheat samples.

Subsequently, we have installed the LED as the induced illuminant to measure the DL signals of wheat samples in five different years, the schematic diagram of which can be seen in Fig. 2. Specifically, taking wheat sample in year of 2015 for an example, the weight of each sample is (25 ± 0.02) g and the total number of samples is 100 groups, and then we put these samples into a dark box for 30 min before testing in order to minimize the influences caused by other stray light. Furthermore, we have designed a set of button switch device to control the lighting time manually. After many attempts, we have selected the lighting time as 60 s, and then measured DL signals of wheat samples in other years under the same working conditions.

### DL signal data preprocessing process

**Figure 3:**
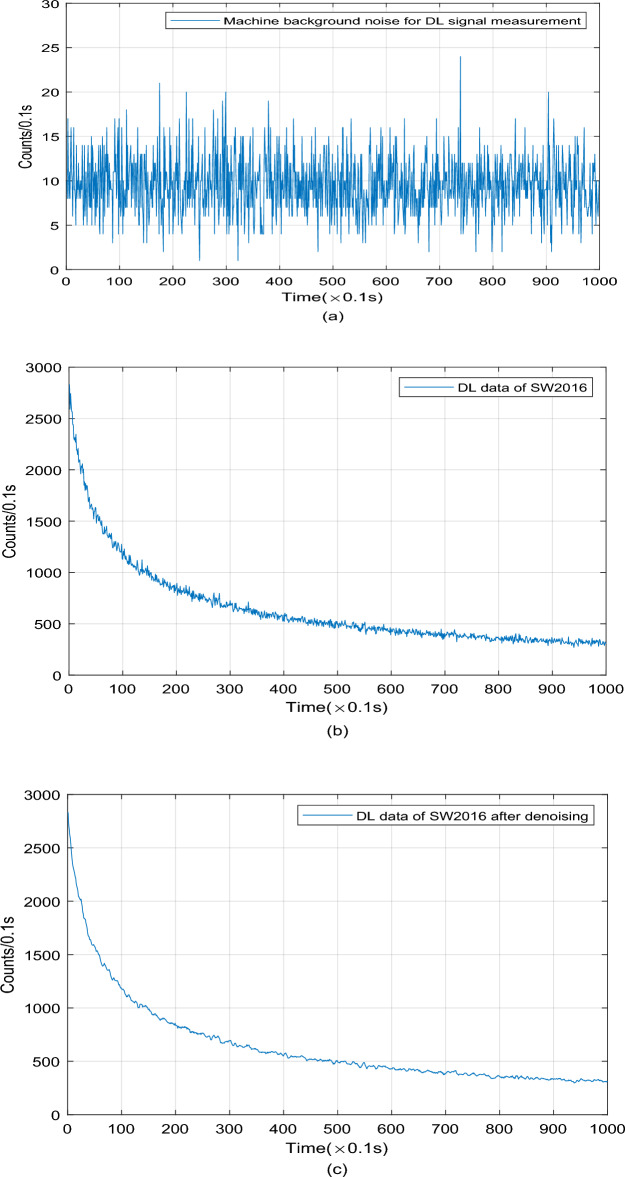
Data preprocessing process to the DL signal of SW2016 wheat sample.

While measuring DL signals of wheat samples, the photon signals are not only influenced by the dark current from other electronic components, but also affected by the changes of environmental conditions. For that reason, the preprocessing to the DL signals is of necessity. We take certain SW2016 sample as an example to show the specific preprocessing process to the DL signal data, which can be seen in Fig. 3.

First, the average background noise intensity (9 counts/0.1 s) of the UWL analyzer is calculated according to the experimental parameters mentioned above, which is seen in Fig. 3a. Second, the signals are collected according to the mode of subtracting the average background noise intensity value, as shown in Fig. 3b. Third, the DL signals obtained in the second step are smoothed and denoised by the sliding average method to get the final sample data, and the sliding window value is set to 5, which is shown in Fig. 3c.

## Classification feature extraction method

In order to better feature DL signals of SW2015–SW2019, we take advantage of following methods to extract ten classification features in this work.

### Hyperbolic relaxation feature extraction method

It has been validated that the organism system induced by a white or other monochromatic illuminants shows a dynamic relaxation behavior, which has the following characteristics:

- show a comparative long relaxation time.
- the end of relaxation process is proximate to SL intensity.
- the whole relaxation process can be fitted by a hyperbolic function perfectly.

**Figure 4:**
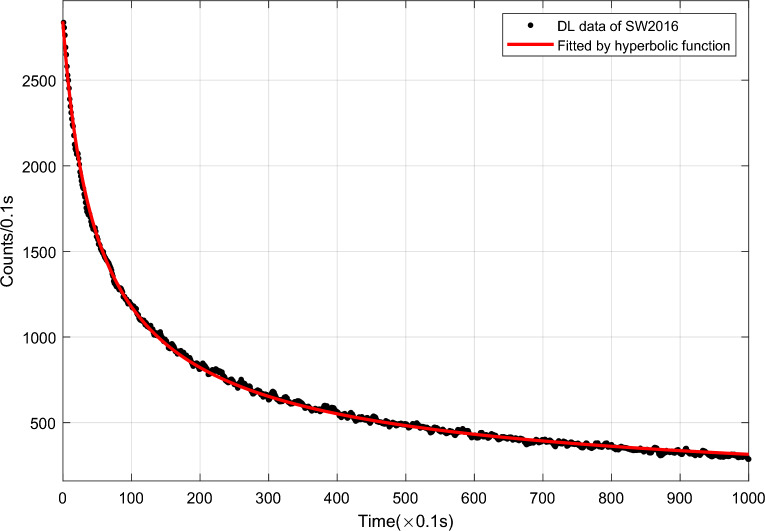
Schematic plots of DL signal of certain sample SW2016 fitted by hyperbolic function.

The detailed hyperbolic function can be described as

1 $$I\left( t \right) = I_{0} /\left( {1 + t/\tau } \right)^{\beta }$$

where $I_{0}$ is the initial luminescence intensity, which depends on the properties of sample as well as lighting conditions; $\tau$ is a time feature, which is only related to the properties of sample; $\beta$ used for controlling the relaxation rate is an exponential factor^22^. Figure 4 shows a schematic plots of DL signal of certain sample SW2016 fitted by hyperbolic function.

Table 1 shows the hyperbolic fitting parameters and evaluation indices of the DL signals of certain samples in five years, where SSE stands for Sum of Squares due to Error, and RMSE is the abbreviation of Root Mean Squared Error, and $R^{2}$ represents Coefficient of Determination. Seen from the evaluation indices in Table 1, the value of RMSE is less than 21 and the $R^{2}$ value is more than 0.99, which validates that the DL signals of wheat samples can be perfectly fitted by certain hyperbolic functions, and lays a foundation for feature extraction to DL signals based on the fitting functions.

**Table 1:** The corresponding fitting parameters and evaluating indices of DL signals in five years.

Wheat sample	$I_{0}$	$\beta$	$\tau$	SSE	RMSE	$R^{2}$
SW2015	2907	0.72	44.47	2.91e+05	17.07	0.9986
SW2016	2837	0.65	34.46	2.48e+05	15.76	0.9985
SW2017	3080	0.66	34.58	3.58e+05	18.93	0.9983
SW2018	3434	0.79	63.08	4.23e+05	20.59	0.9987
SW2019	2876	0.74	45.63	2.33e+05	15.27	0.9989

Moreover, we can also resort to a time window to calculate the integral calculus of (1) with the range of [0,T], which is shown as in formula (2)

2 $$E(T) = \int_{0}^{T} {\frac{{I_{0} }}{{(1 + t/\tau )^{\beta } }}dt = \frac{{\tau I_{0} }}{\beta - 1} \left [1 - \frac{1}{{(1 + T/\tau )^{\beta - 1} }} \right]}$$

where, $T$ is the measuring time. $E(T)$ stands for the calculus intensity of the DL with the range of $[0,T]$, and the value of $E(T)$ is depended on the parameters $I_{0} ,\tau ,\beta$ and $T$.

Thus, to the DL data of wheat samples, we can use above-mentioned four parameters ($I_{0}$, $\beta$, $\tau$ and $E(T)$) as part of classification features to represent the relaxation characteristics of DL signals.

### Other feature extraction methods

Besides the above-mentioned four features, we also elaborately choose the following six features to shape the final classification feature vector to feature the DL signals.

#### Instantaneous frequency

Instantaneous frequency (IF) of a time sequence signal is that the mean of frequencies $f$ in the signal changes with diverse time instant parameters $t$, which provides a measure of signal energy concentration in the frequency domain as a time function^23,24^. Thus the function IF(t) can be used to estimate the IF of a signal at instant time $t$ through computing (3)

3 $$IF(t) = \frac{{\int_{ - \infty }^{ + \infty } {fP(t,f)df} }}{{\int_{ - \infty }^{ + \infty } {P(t,f)df} }}$$

where $P(t,f)$ is the signal power spectrum.

In this work, due to the DL signal is measured in a time interval $0 \le t \le T$ rather than in an infinite length. For this reason, we take advantage of a special method for computing the power spectrum of time sequence $x_{k} ,k = 0,1,...,N - 1$, and then the power spectral density of $x_{k}$ can be approximately described as^25^.

4 $$P_{N} (f) = \frac{\Delta t}{N}\left| {\sum\limits_{k = 0}^{N - 1} {x_{k} e^{ - 2\pi ifk\Delta t} } } \right|^{2}$$

where $\Delta t$ stands for the sampling interval.

We can rewrite (4), assuming $f = j\Delta f$, $\Delta f = 1/(N\Delta t)$, $\Delta t$ = “1”, and then we can obtain

5 $$P_{j} = \frac{1}{N}\left| {\sum\limits_{k = 0}^{N - 1} {x_{k} e^{{ - 2\pi i\frac{jk}{N}}} } } \right|^{2} = \frac{1}{N}\left| {X_{k} } \right|^{2}$$

where $X_{k}$ is the discrete Fourier transform (DFT) of $x_{k}$, which can be seen as follows.

6 $$X_{j} = \sum\limits_{k = 0}^{N - 1} {x_{k} e^{{ - 2\pi i\frac{jk}{N}}} }, \quad j = 0,1,\;...,N - 1$$

In order to fully use the power spectrum characteristics, we resort to the Kaiser window to scale $P_{j}$. Thus (5) can be rewritten as

7 $$P_{j} = \frac{1}{WN}\left| {\sum\limits_{k = 0}^{N - 1} {w_{k} x_{k} e^{{ - 2\pi i\frac{jk}{N}}} } } \right|^{2}, \quad j = 0,1,...,N - 1$$

Among which

8 $$W = \frac{1}{N}\sum\limits_{j = 0}^{N - 1} {w_{j}^{2} }, \quad j = 0,1,...,N - 1$$

where $w_{j}$ is the weight of the Kaiser window function.

So the $P_{j}$ in (7) can be calculated by the following steps:

- In order to simplify the computation process, the length of initial time series is taken ${\text{N = 2}}^{n}$, if not, padding that with zeros, $n$ stands for a positive integer.
- Use a corresponding window function $w_{k}$ to weight the above series.
- Resort to the fast Fourier transform (FFT) to calculate the discrete Fourier transformation (DFT) of the weighted series $w_{k} x_{k}$.
- Calculate $$P_{j}$$ by (7).

#### Spectral entropy

Spectral entropy (SE) is used to measure the signal spectral power distribution^26^. Normally, there remain two steps for calculating the SE at time $t$, which is denoted as $H(t)$. Firstly, assume the probability distribution of a time–frequency power spectrum as $P(t,f)$, and $p(t,m)$ stands for frequency point $m,m = 1,2,...,N$, which can be calculated by

9 $$p(t,m) = \frac{P(t,m)}{{\sum\limits_{f} {P(t,f)} }}$$

where $f \in [0,f_{s} /2]$, $f_{s}$ denotes the sampling frequency, and we take $f_{s}$ = 10 HZ in this work due to the sampling time is 0.1 s. Secondly, according to the computation process of Shannon entropy, the spectral entropy $H(t)$ can be obtained by

10 $$H(t) = - \sum\limits_{m = 1}^{N} {p(t,m)\log_{2} p(t,m)}$$

#### Approximate entropy

The approximate entropy (ApEn) algorithm was initially proposed by Pincus, aiming at measuring the properties of random series^27^. Two striking advantages of the ApEn lies in its lower dependency on the length of initial time series and robust resistance to the noise carried by the initial data. The detailed computing process of the ApEn algorithm is:

1. Partition the initial series $X = \{ x(i),i = 1,2,...,N\}$ into an m-dimensional vector $u(i)$, which is described as
  11 $$u(i) = \{ x(i),x(i + 1),...,x(i + m - 1)\} , \quad i = 1,2,...,N - m + 1$$
  Here, $m$ stands for the dimension of the pattern vector, and $N$ is the initial length of the time series.
2. Calculate the distance $d[u(i),u(j)]$ between vector $u(i)$ and vector $u(j)$ using (12).
  12 $$d[u(i),u(j)] = \mathop {\max }\limits_{k = 0,1,...,m - 1} |x(i + k) - x(j{ + }k)|$$
3. Count the numbers of $d[u(i),u(j)] < r$, where $r$ is a positive real number, which is known as the similar tolerance threshold value. Consequently, calculate the proportion of $d[u(i),u(j)] < r$ among the total number of vectors, which is labeled as $C_{i}^{m} (r)$
  13 $$C_{i}^{m} (r) = (numbe \; r\left. {of \; \left. {d[u(i),u(j)] < r} \right)/(N - m + 1)} \right\})$$
4. Calculate the logarithm of $C_{i}^{m} (r)$, and then, obtain its mean value by (14). Here, the mean value is labeled as $H^{m} (r)$.
  14 $$H^{m} (r) = \frac{1}{N - m + 1}\sum\limits_{i = 1}^{N - m + 1} {\ln C_{i}^{m} (r)}$$
5. By increasing the dimension from $m$ to $m + 1$ and repeating above steps 2–4, we can obtain $H^{m + 1} (r)$.
  Then the definition of the ApEn can be described as
  15 $$ApEn(m,r) = \mathop {\lim }\limits_{N \to \infty } [H^{m} (r) - H^{m + 1} (r)]$$
  If $N$ is finite, formula (15) can be rewritten as follows:
  16 $$ApEn\left( {m,r,N} \right) = H^{m} \left( r \right) - H^{m + 1} \left( r \right)$$
  From the above formulas, we can deduce that the ApEn value is prone to be lager provided that the initial series is more complex. Thus, the ApEn can be used as a classification feature to characterize the DL signals of wheat samples.

#### Median

Rearranging the measured DL data of a sample in a descend order, the value in the middle of the arranged sequence is the median. Namely, in the entire sequence, the number of less than the median and greater than the median is equivalent. If the total number is even, take the average of the most middle two values as the median.

#### Quartile deviation

Quartile is a set of data in a descend number, using three points to divide the whole data into four equal parts. We mark the corresponding three division points by Q1,Q2 and Q3 respectively. Q1 (the first quartile) indicates 25% of the data less than or equal to Q1; Q2 (the second quartile, namely median) means 50% of the data is less than or equal to Q2; Q3 (the third quartile) points 75% of the data is less than or equal to Q3. Among them, the distance between Q3 and Q1 is also called quartile deviation (QD), denoted by QD = Q3-Q1. The QD reflects the dispersion degree of the middle 50% data, and the smaller of QD reflects the more concentrated the data in the middle is, and vice versa. Sometimes, we will resort to interpolation method to calculate the value of Q1 and Q3, provided that the number of (n + 1) can not be divisible by four, where n is the total sampling points.

#### Mean deviation

Mean deviation (MD) can be calculated by the following equation:

17 $$MD = \frac{{\sum\nolimits_{i = 1}^{N} {\left| {x_{i} - \mu } \right|} }}{N}$$

Here $N$ is the total sampling points, and $\mu$ is the corresponding mathematical expectation.

The value of MD characterizes the differences degree between each sample point and the arithmetic mean. The greater value shows the smaller representation of the arithmetic mean and vice versa.

## Wheat aging detection model design

### Bi-LSTM network

**Figure 5:**
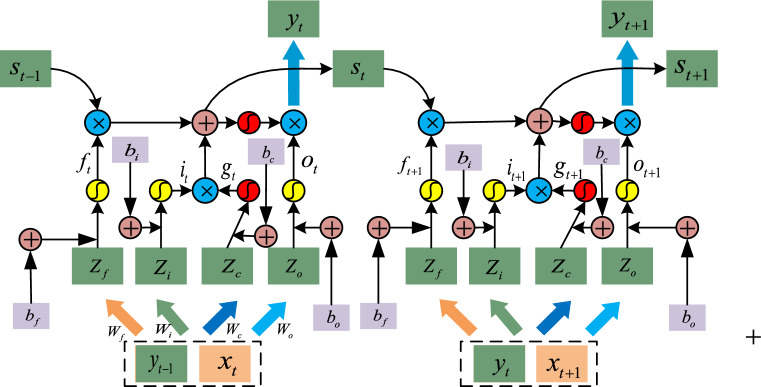
The detailed inner structure of LSTM unites.

Long Short-term Memory (LSTM), proposed by Hochreiter and Schmidhuber^28^, is a special Recurrent Neural Networks (RNN), which is capable of handling long-term dependencies as well as solving the gradient vanishing problem existed in conventional RNNs. To the LSTM network, it takes advantage of diverse gates shaped by input data multiply different vectors to control data flows. Concretely, the input data controls how much the new data can be used in the current memory cell; meanwhile, the forget gate makes a decision that how much data should be removed from the former memory cell; finally, the output gate decides how much data will be output from the current memory cell. The detailed inner structure of LSTM units is shown in Fig. 5, and the red activation function stands for hyperbolic tangent function while the yellow activation function is sigmoid function. By virtue of these diverse function gates, LSTM network is capable of processing time sequences effectively to achieve the purpose of classification or prediction.

To initial DL data, we resort to a zero mean normalization method (Z-score standardization) to normalize the initial data. The normalization equation can be described as

18 $$\hat{x}_{i} = \frac{{x_{i} - \mu }}{\sigma }$$

where $x_{i} (1 \le i \le t)$ stands for all the elements of the input vector, $\mu { = }\frac{1}{t}\sum\nolimits_{i = 1}^{t} {x_{i} }$ and $\sigma { = }\sqrt {\frac{1}{t - 1}\sum\nolimits_{i = 1}^{t} {(x_{i} - \mu )^{2} } }$ represent its corresponding mean and sample standard deviation respectively.

Consequently, we take advantage of LSTM network to train the labeled normalization DL data $\hat{x} = (\hat{x}_{1} ,\hat{x}_{2} ,...,\hat{x}_{t} )$, and the whole process can be described by the following formulas according to Fig. 5

19 $$i_{t} = \sigma (W_{i} \cdot [y_{t - 1} ,x_{t} ] + b_{i} )$$

20 $$f_{t} = \sigma (W_{f} \cdot [y_{t - 1} ,x_{t} ] + b_{f} )$$

21 $$o_{t} = \sigma (W_{o} \cdot [y_{t - 1} ,x_{t} ] + b_{o} )$$

22 $$g_{t} = \tanh (W_{c} \cdot [y_{t - 1} ,x_{t} ] + b_{c} )$$

23 $$s_{t} = f_{t} \odot s_{t - 1} + i_{t} \odot g_{t}$$

24 $$y_{t} = o_{t} \odot \tanh (s_{t} )$$

where $W_{*}$ stands for the corresponding matrix weights;$b_{*}$ denotes the different bias scalars; $\sigma ( \cdot )$ is sigmoid function; $\tanh ( \cdot )$ represents hyperbolic tangent function. $i,f,o,g,s$ are input gate, forget gate, output gate, candidate values, and new cell state successively; $y_{t}$ refers to the final output and $\odot$ means the element-wise product between two vectors.

Softmax Classifier: the softmax function $s{\text{ = [s}}_{1} {\text{,s}}_{2} {,}...{\text{,s}}_{M} {]}$ can be defined as (25), which maps M input data vector into M normalized output data.

25 $$s_{i} = \frac{{e^{{k_{f}^{T} }} w_{i} }}{{\sum\nolimits_{m = 1}^{M} {e^{{k_{f}^{T} w_{m} }} } }}\begin{array}{*{20}c} {} & {i = 1,2,...,M} \\ \end{array}$$

where $w_{i}$ stands for the weight vector of the fully connected layer, $k_{f}$ refers to the final output in the second layer, $( \cdot )^{T}$ means transpose operation.

In order to train the weights of LSTM network and measure the difference between the normalized output and the true label, we have introduced cross entropy as the loss function $L(w)$, and adopted $L2$ regularization method so as to prevent over-fitting and improve the generalization performance of network, which can be describes as

26 $$L(w) = - \sum\limits_{i = 1}^{M} {y_{i} \log (s_{i} ) + \frac{\lambda }{2N}\sum\limits_{j = 1}^{N} {w_{i}^{{2}} } }$$

where $y_{i}$ denotes for the true labeled data for the concrete wheat storage year, and $w_{i}$ is the weight parameter, and $\lambda \in R$ represents the regularization coefficient in order to adjust weight between regularization term and original loss value. Meanwhile, $M$ stands for the categories of output of softmax classifier, and $N$ is the number of training set.

To the error term of LSTM, it has been involved in two directions: one way is that we can compute it along the time dimension by back propagation, namely, computing every error term before $t$ moment; the other way is transmitting the error to the front layer. However, fewer researchers pay attention the second one, so we mainly resort to the first method to calculate the error term, which can be described as

27 $$\delta_{t}^{l - 1} = \frac{\partial J}{{\partial h_{t}^{l - 1} }} = \delta_{i,t}^{l} w_{ih} + \delta_{f,t}^{l} w_{fh} + \delta_{g,t}^{l} w_{gh} + \delta_{o,t}^{l} w_{oh}$$

where $\delta_{i,t}^{l} ,\delta_{f,t}^{l} ,\delta_{g,t}^{l}$ and $\delta_{o,t}^{l}$ are standing for the error term of input gate, forget gate, candidate value and output gate in $l^{th}$ layer respectively. Furthermore, we can deduce them by following formulas.

28 $$\delta_{i,t}^{l} = \delta_{t}^{l} \cdot o_{t}^{l} \cdot f{\prime} (c_{t}^{l - 1} ) \cdot g_{t}^{l} \cdot i_{t}^{l} \cdot (1 - i_{t}^{l} )$$

29 $$\delta_{f,t}^{l} = \delta_{t}^{l} \cdot o_{t}^{l} \cdot f{\prime} (c_{t}^{l} ) \cdot c_{t - 1}^{l} \cdot f_{t}^{l} \cdot (1 - f_{t}^{l} )$$

30 $$\delta_{o,t}^{l} = \delta_{t}^{l} \cdot f{\prime} (c_{t}^{l} ) \cdot o_{t}^{l} \cdot (1 - o_{t}^{l} )$$

31 $$\delta_{g,t}^{l} = \delta_{t}^{l} \cdot o_{t}^{l} \cdot f{\prime} (c_{t}^{l} ) \cdot i_{t}^{l} \cdot (1 - (g_{t}^{l} )^{2} )$$

Here, $f^{\prime} ( \cdot )$ means the differential of active function, and we always choose tanh and sigmoid function as active function in LSTM network. Thus, each element in weight matrix is less than one, and we can see from the above formula that there remain positive or negative signs among the four terms, which makes the value of $\delta_{t}^{l - 1}$ decrease gradually and causes the problem of gradient vanishment.

After taking all the above-mentioned factors into consideration, we finally resorted to the Back Propagation Through Time (BPTT) algorithm to train the parameters of LSTM network based on the above loss function^29^, in which Adam Optimizer was used for the sake of making the computation process more efficient^30^.

**Figure 6:**
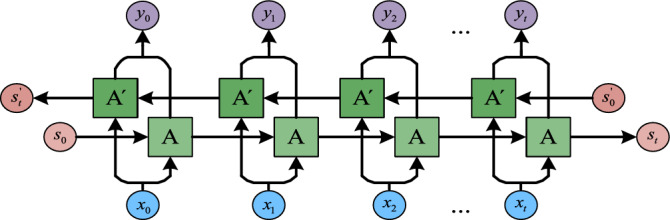
The structure of Bi-LSTM network.

In order to improve the classification performance of the proposed model, we have introduced a bidirectional LSTM (Bi-LSTM) network to learn the “future information” of input sequence^31^. Figure 6 shows the concrete Bi-LSTM network structure, among which $\{ x_{0} ,x_{1} ,x_{2} ,...,x_{t} \}$ and $\{ y_{0} ,y_{1} ,y_{2} ,...,y_{t} \}$ are the input and the output sequence respectively; A and Á stand for the respective LSTM units; $s_{0}$,$s_{t}$,$s_{0}{\prime}$ and $s_{t}{\prime}$ are the different cell states. This type of network not only can be trained in two directions simultaneously, but also has the separate hidden layers.

### Mechanism of encoder-decoder based on Walsh code

In order to make the classification process more robust, we resort to Walsh code to generate the labeled output sequences in this paper. There exist several methods for producing Walsh code sequence^32^, and we mainly take advantage of Hadamard matrix to generate Walsh code in this work. The concrete steps can be seen as follows: we assume $H_{N}$ denotes the Hadamard matrix with dimension $N \times N$, among which any two rows or columns are orthogonal. Namely, if we regard the row or column in $H_{N}$ as a function, the values of cross correlative function between them are zeros. Walsh code can be generated by the following recursive procedure, which can be describes as

32 $$H_{2N} = \left[ {\begin{array}{*{20}c} {H_{N} } & {H_{N} } \\ {H_{N} } & {\overline{{H_{N} }} } \\ \end{array} } \right]$$

Here $N = 2^{n} ,n \in N^{ + }$,$\overline{{H_{N} }}$ is the logical reverse of $H_{N}$, and normally we set $H_{1} = [0][0]$.

Since our final classification targets in this paper are five, we take $N = 8$ to generate the target sequences, which can be described as

33 $$H_{2} = \left[ {\begin{array}{*{20}c} {H_{1} } & {H_{1} } \\ {H_{1} } & {\overline{{H_{1} }} } \\ \end{array} } \right] = \left[ {\begin{array}{*{20}c} 0 & 0 \\ 0 & 1 \\ \end{array} } \right], \ldots ,H_{8} = \left[ {\begin{array}{*{20}c} {H_{4} } & {H_{4} } \\ {H_{4} } & {\overline{{H_{4} }} } \\ \end{array} } \right] = \left[ {\begin{array}{*{20}c} 0 & 0 & 0 & 0 & 0 & 0 & 0 & 0 \\ 0 & 1 & 0 & 1 & 0 & 1 & 0 & 1 \\ 0 & 0 & 1 & 1 & 0 & 0 & 1 & 1 \\ 0 & 1 & 1 & 0 & 0 & 1 & 1 & 0 \\ 0 & 0 & 0 & 0 & 1 & 1 & 1 & 1 \\ 0 & 1 & 0 & 1 & 1 & 0 & 1 & 0 \\ 0 & 0 & 1 & 1 & 1 & 1 & 0 & 0 \\ 0 & 1 & 1 & 0 & 1 & 0 & 0 & 1 \\ \end{array} } \right]$$

We can obviously see from (33), either the $i{\text{th}}$ row or the $i{\text{th}}$ column ($i$ is row or column number of the matrix) in $H_{8}$ shows the same coding pattern, which not only exhibits a strong robust ability, but also easily extends according to the requirement. In this work, we successively select the first five rows or columns as the representations of the corresponding wheat sample labels from 2015 to 2019.

Since we have adopted Walsh code as the output sequences, an encoder-decoder mechanism for the corresponding labels is introduced in the classification model. We assume the input and the output sequence of encoder are $$Y_{t} = \{ y_{1} ,y_{2} ,...,y_{t} \}$$ and $$\hat{Y}_{s} = \{ \hat{y}_{1} ,\hat{y}_{2} ,...,\hat{y}_{2N} \}$$ respectively. $$t$$ is the length of the one-hot vector for representing wheat aging labels, and $$N$$ is the dimension size of Hadamard matrix. We take wheat sample in 2015 for an example, the label vector before and after encoder is written as $$Y_{t} = \{ 1,0,0,0,0\}$$ and $$\hat{Y}_{s} = \{ 0,0,0,0,0,0,0,0\}$$ separately. Normally we take $$N = 2^{n} \approx t$$$$\left( {n \in N^{ + } } \right)$$. Reversely, in the decoder part, we can directly use $$\hat{Y}_{s}$$ to serve as the inputs of the decoder to obtain the output sequence $$Y_{t}$$. Seen from the computer simulation results in later sections, though introducing Walsh code and encoder-decoder mechanism, the anti-noise ability and classification accuracy of the model will be improved obviously.

### Walsh-Bi-LSTM network

**Figure 7:**
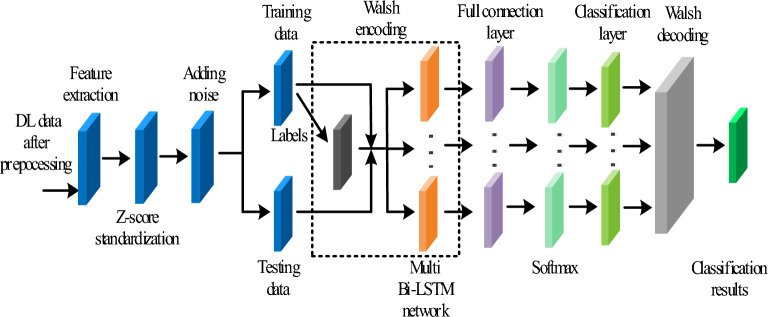
The whole flowchart of the detection model for wheat aging.

As the Bi-LSTM network lacks error-correcting performance, we propose the Walsh-Bi-LSTM network to establish the final detection model, aiming at making the detection model have the error-correcting performance by reasonably splitting the multi-classification target task. The whole flowchart of the detection model is shown in Fig. 7. Moreover, in order to test the error-correction performance of the model, noise is added to all data before entering the model, where the dashed box represented the Walsh encoding module.

## Experiment results

In this part, we carried out comparable experiments to validate the classification performance of our proposed model. In order to make a comparison, we tested the wheat aging classification performance by the normal Bi-LSTM network and the Walsh-Bi-LSTM respectively. The performance of classification model was assessed by three indices: training accuracy curve, training loss curve and confusion chart.

### Datasets

We carried out all the experiments based on the DL data of wheat samples stored in five different years. First of all, we take advantage of above-mentioned feature extraction methods to obtain the final datasets. In addition, the datasets of each type were divided into two groups, namely, training group (80 samples) and testing group (20 samples), and each sample was marked by the corresponding classification labels.

### Implementation details

In this work, we adopted normal Bi-LSTM network and Walsh-Bi-LSTM as the detection models respectively for the sake of making a comparison between their classification performance. After several tests, we finally chose the following hyperparameters: The batch size was 8; the number of epochs was 100; learning rate was 5e−4; hidden layers was 128 and Adam optimizer was adopted. To the Walsh-Bi-LSTM, we took $H_{8}$ to encode and decode the output sequences, among which we chose the top five rows or columns as the representation of corresponding five classification labels. Furthermore, we used the ten above-mentioned features extracted from DL signal as the training and testing data for two networks. Subsequently, both of networks was implemented by TensorFlow framework and trained on the basis of NVIDIA GeForce GTX 1060 3 GB.

### Classification evaluation of Bi-LSTM network and Walsh-Bi-LSTM network

In this part, we have trained the Bi-LSTM network and the Walsh-Bi-LSTM network separately and made a classification performance evaluation between them. Seen from Fig. 7, we have taken z-score operations and added noise so as to test the robust performance of classification models.

**Figure 8:**
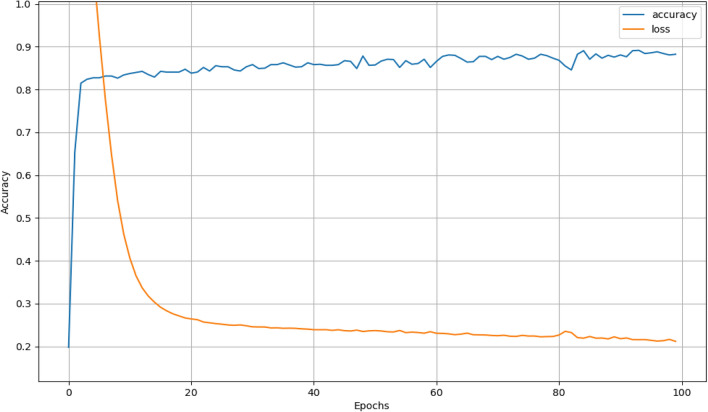
The training accuracy and loss curve of Bi-LSTM network.

**Figure 9:**
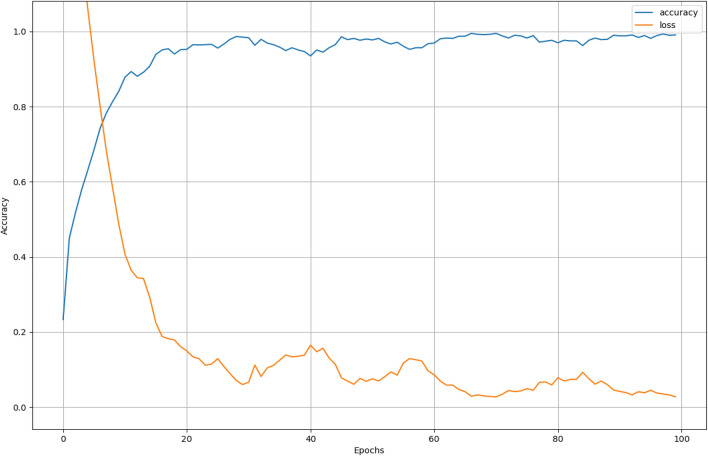
The training accuracy and loss curve of the Walsh-Bi-LSTM network.

**Figure 10.**
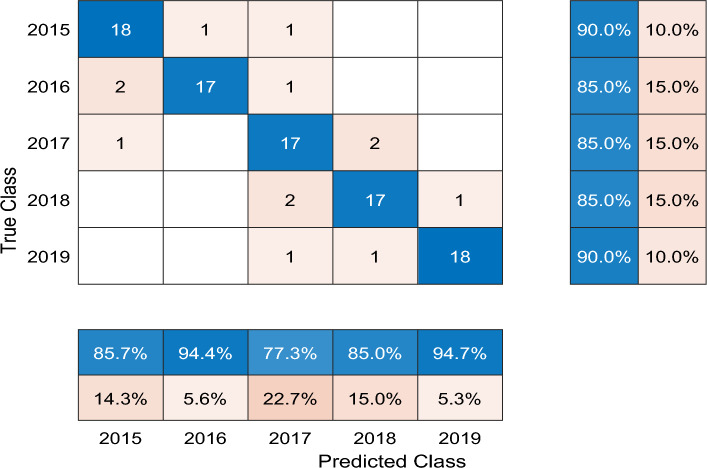
The confusion chart of Bi-LSTM network.

**Figure 11:**
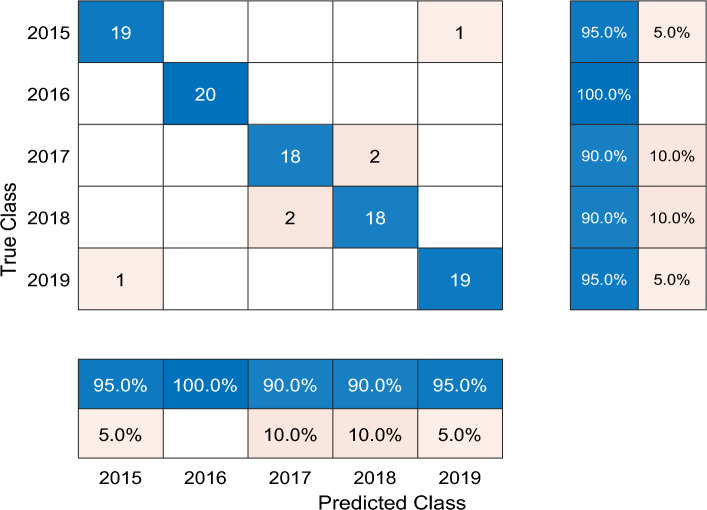
The confusion chart of the Walsh-Bi-LSTM network.

After simulating by computer, the training average accuracy of the Bi-LSTM network and the Walsh-Bi-LSTM network is 87.3% and 99.6% respectively, which can be seen in Figs. 8 and 9. To the testing dataset, the average accurate classification rate of the Bi-LSTM is 87.0%, and the misclassification results mainly scatter in the adjacent years due to the one-hot vector used as final output labels, which can be seen in Fig. 10. Meanwhile, the average accurate classification rate of the Walsh-Bi-LSTM in the same testing dataset is 94.0%, and the misclassification results randomly distribute in other years due to the Walsh encoding mechanism, which can be seen in Fig. 11.

Experimental results show that the average training classification accuracy and testing classification accuracy of Walsh-Bi-LSTM network achieve 99.6% and 94% respectively, which have been improved by 12.3% and 7.0% compared with Bi-LSTM network. Moreover, the Walsh-Bi-LSTM network can be trained well in a much shorter time compared with the normal Bi-LSTM network. Therefore, the experiment result validates that our proposed method is feasible and practical for classifying the aging degree of wheat samples.

### Classification results compared with other methods

In order to better show the classification performance of our proposed method, we have made a comparison with some representative classification methods. The detailed classification results of different models based on the same features mentioned above can be seen in Table 2.

**Table 2:** The classification results of different models based on the same features.

Model name	Average training classification accuracy (%)	Average testing classification accuracy (%)
SVM (Support Vector Machine)	82.13	74.00
GNB (Gaussian Naive Bayes)	85.63	78.00
LDA (Linear Discriminant Analysis)	78.50	69.00
DT (Decision Tree)	90.38	72.00
Bi-LSTM network	87.30	87.00
Walsh-Bi-LSTM network	99.60	94.00

Seen from Table 2, we observe that the Bi-LSTM network can obtain a better classification results both in training set and testing set, and the main reason is that DL signals of wheat kernels have strong time association features. Moreover, it is also validated that Walsh-Bi-LSTM network proposed in this work does have error-correcting performance, which is a feasible and reliable way to detect wheat aging degree.

## Conclusions

We have proposed a set multi-class wheat aging detection model using the Walsh-Bi-LSTM network based on DL signals of wheat kernels. Shown by the experiment results, the DL signals of wheat kernels can be used as an index to reflect their inner physiological activity like wheat aging. The detection technique to wheat aging based on DL is an eco-friendly and nondestructive method. Moreover, compared with detection methods based on SL signals, the detection methods based on DL signals have a higher SNR as well as easily extract classification features. Since DL signal obeys certain hyperbolic function decay trend, we can resort to Bi-LSTM network and Walsh-Bi-LSTM network to establish the classification model respectively after obtaining enough data samples. The simulation results show that the Walsh-Bi-LSTM network is prone to be more efficient and feasible in detecting wheat aging degree. Our next work is establishing a model for predicting the wheat aging states, which can help granary managers to evaluate the stored wheat aging states and make scientific decisions.

## Data Availability

The datasets generated and/or analysed during the current study are not publicly available due all the samples used in this work from the national grain depot but are available from the corresponding author on reasonable request.
